# A Randomized Comparison of Chloroquine versus Dihydroartemisinin–Piperaquine for the Treatment of *Plasmodium vivax* Infection in Vietnam

**DOI:** 10.4269/ajtmh.15-0740

**Published:** 2016-04-06

**Authors:** Phung Duc Thuan, Nguyen Thuy Nha Ca, Pham Van Toi, Nguyen Thanh Thuy Nhien, Ngo Viet Thanh, Nguyen Duc Anh, Nguyen Hoan Phu, Cao Quang Thai, Le Hong Thai, Nhu Thi Hoa, Le Thanh Dong, Mai Anh Loi, Do Hung Son, Tran Tinh Ngoc Khanh, Christiane Dolecek, Ho Thi Nhan, Marcel Wolbers, Guy Thwaites, Jeremy Farrar, Nicholas J. White, Tran Tinh Hien

**Affiliations:** Oxford University Clinical Research Unit, Ho Chi Minh City, Vietnam; Institute of Malaria-Parasitology-Entomology, Ho Chi Minh City, Vietnam; Oxford University Global Health MSc Program, Oxford University, Oxford, United Kingdom; Centre for Tropical Medicine and Global Health, Oxford University, Oxford, United Kingdom; Mahidol-Oxford Tropical Medicine Research Unit, Faculty of Tropical Medicine, Mahidol University, Bangkok, Thailand

## Abstract

A total of 128 Vietnamese patients with symptomatic *Plasmodium vivax* mono-infections were enrolled in a prospective, open-label, randomized trial to receive either chloroquine or dihydroartemisinin–piperaquine (DHA-PPQ). The proportions of patients with adequate clinical and parasitological responses were 47% in the chloroquine arm (31 of 65 patients) and 66% in the DHA-PPQ arm (42 of 63 patients) in the Kaplan–Meier intention-to-treat analysis (absolute difference 19%, 95% confidence interval = 0–37%), thus establishing non-inferiority of DHA-PPQ. Fever clearance time (median 24 versus 12 hours, *P* = 0.02), parasite clearance time (median 36 versus 18 hours, *P* < 0.001), and parasite clearance half-life (mean 3.98 versus 1.80 hours, *P* < 0.001) were all significantly shorter in the DHA-PPQ arm. All cases of recurrent parasitemia in the chloroquine arm occurred from day 33 onward, with corresponding whole blood chloroquine concentration lower than 100 ng/mL in all patients. Chloroquine thus remains efficacious for the treatment of *P. vivax* malaria in southern Vietnam, but DHA-PPQ provides more rapid symptomatic and parasitological recovery.

## Introduction

*Plasmodium vivax* is the second most prevalent malaria in the world. It is estimated that 2.85 billion people live at risk of infection.^1^ *Plasmodium vivax* is endemic throughout the tropics except for much of sub-Saharan Africa. The global burden of *P. vivax* malaria is estimated to range from ∼70 to 80 million cases annually, with about 80–90% of cases occurring in the Middle East, Asia, and the Western Pacific.^2^

Chloroquine-resistant *P. vivax* has been documented in Columbia, Peru, Bolivia, Brazil, Guyana, Turkey, Ethiopia, Madagascar, India, Myanmar, Thailand, Vietnam, Indonesia, Papua New Guinea, and South Korea.^3^ *Plasmodium falciparum* and *P. vivax* are the most widespread malaria species in Vietnam, representing 61% and 34% of all cases, respectively.^4^ Chloroquine remains the first-line treatment of *P. vivax* malaria in all countries except Indonesia, Papua New Guinea, the Solomon Islands, Vanuatu, and Cambodia where artemisinin combination therapies (ACTs) have been adopted since 2009.^5^,^6^ Concurrent radical treatment with primaquine improves the activity of chloroquine against resistant blood-stage parasites.^7^,^8^ Recurrences of *P. vivax* on or before day 28, or prophylactic failure, has been observed in Asia, Africa, and Latin America.^9^ Previous studies indicated that chloroquine has high efficacy for the treatment of *P. vivax* in Vietnam.^10^,^11^ However, in a study from 1997 to 2000 in Binh Thuan Province, southern Vietnam, 16% chloroquine resistance at level I (RI) of *P. vivax* was detected.^12^ The prevalence of chloroquine-resistant *P. vivax* in other provinces in Vietnam such as Ninh Thuan and the Central, is of concern.^13^,^14^

Recent worsening of antimalarial drug resistance of *P. falciparum* malaria in southeast Asia^15^ indicates the need to assess the efficacy of artemisinin and its derivatives in patients with *P. vivax* malaria. Dihydroartemisinin–piperaquine (DHA-PPQ) is the first-line treatment of *P. falciparum* malaria in Vietnam. Clinical studies have shown that DHA-PPQ clears *P. vivax* infections more quickly than chloroquine.^16^,^17^ Lower recurrence rates with DHA-PPQ have also been observed in Afghanistan, Thailand, and Indonesia.^16^–^18^ To reassess the efficacy of chloroquine in the treatment of *P. vivax* infections and to evaluate the efficacy of DHA-PPQ, we conducted a randomized controlled trial in patients with *P. vivax* mono-infections in Vietnam.

## Materials and Methods

### Study sites and patients.

The study took place at Bu Gia Map and Dak O communes of Bu Gia Map District, Binh Phuoc Province, an area of high malaria transmission in Vietnam. Patients ≥ 3 years old with uncomplicated vivax malaria were eligible for enrollment if they had mono-infection with parasitemia ≥ 250/μL asexual forms, had axillary or tympanic temperature ≥ 37.5°C (or history of fever during the past 24 hours) and if they or their guardians gave fully informed consent. Patients were excluded if they had febrile conditions due to diseases other than malaria or other known underlying chronic or were severely ill, were on regular medications that may interfere with antimalarial pharmacokinetics, had received antimalarial drug in the previous 48 hours, had a history of hypersensitivity or contraindications to study drugs, had splenectomy, or were in the first trimester of pregnancy.

### Study design and randomization.

This study was a prospective open-label randomized comparison of the efficacy of DHA-PPQ versus chloroquine in *P. vivax* malaria.^19^,^20^ Patients who presented to health stations of Dak O and Bu Gia Map communes, gave fully informed written consent, and met the study inclusion criteria were enrolled, screened, and randomized to receive either DHA-PPQ or chloroquine for 3 days, then followed up for 63 days. The follow-up consisted of a fixed schedule of checkup visits and corresponding clinical and laboratory examinations. Randomization was based on a computer-generated randomization list, which used block randomization with a 1:1 allocation ratio, no stratification, and variable block lengths of four and six. Individual randomization assignments were stored in opaque, sealed envelopes. Envelopes were opened in strict numerical sequence and monitored for compliance with the randomization sequence and procedures.

### Drugs.

Patients were randomized to receive either chloroquine (25 mg base/kg for 3 days) or DHA-PPQ (dihydroartemisinin 40 mg + piperaquine phosphate 320 mg per tablet) for 3 days; doses depend on body weight according to National Guidelines.^21^ When the study started, primaquine was not generally available and so was not given routinely. However, standard radical curative 2-week course of primaquine was given to study patients at day 63 or earlier if the patients had a recurrent malaria episode.

DHA-PPQ was sourced from OPC pharmaceutical company (Ho Chi Minh City, Vietnam) (under the brand name CV Artecan) in Vietnam and chloroquine was provided by the Institute of Malaria-Parasitology-Entomology as part of the National Malaria Program.

### Drug measurements.

Chloroquine and monodesethylchloroquine (MCQ) were measured in both whole blood and plasma of patients on the day of enrollment, days 7, 28 and at the time of recurrent parasitemia. Drug levels were determined by high-performance liquid chromatography (HPLC). In brief, the liquid chromatography system was a LaChrom Elite (Merck–Hitachi, Tokyo, Japan) controlled by EZChrom Elite version 3.18 HPLC System Manager Software (Merck–Hitachi). Solid phase extraction (SPE) on Isolute-96-CBA (Biotage AB, Uppsala, Sweden) was used to process 100 μL of plasma/whole blood samples. After the SPE procedure, 50 μL of reconstituted solution was injected into HPLC system. Chloroquine, MCQ, and quinine (internal standard) were separated using a mobile phase consisting of phosphate buffer 25 mM (pH 2.60) and acetonitrile (88:12, v/v) with 2 mM sodium perchlorate on a ZORBAX SB-CN 150 × 4.6-mm, 5-μm column equipped with 5-μm guard cartridges ZORBAX SB-CN 12.5 × 4.6 mm (Agilent Technologies, Santa Clara, CA) at a flow rate of 1.2 mL/minute at ambient temperature in 10 minutes. The retention time of chloroquine was 4.62 minutes, MCQ was 5.95 minutes, and that of quinine (internal standard) was 7.50 minutes. The diode array detector was set at a wavelength of 343 nm. The method was linear over the range of 10–5,000 ng/mL for both chloroquine and MCQ in plasma and whole blood with *r*^2^ > 0.99. The limit of detection was 4 ng/mL and limit of quantification was 10 ng/mL in both plasma and blood for both chloroquine and MCQ. The intra-, inter- and total assay precisions were less than 10% for chloroquine and MCQ in plasma and whole blood samples. In plasma, the accuracies varied between 101% and 103% for chloroquine and MCQ; whereas in whole blood, the accuracies ranged from 97.0–102% for chloroquine and MCQ. The mean recoveries of chloroquine and MCQ were 89.58–91.24% and 84.22–91.32% for plasma and 77.74–82.06% and 75.88–79.76% for blood, respectively. Furthermore, the recovery of quinine (internal standard) in all validation batches was 90.19% (relative standard deviation = 2.46%) for plasma and 91.05% (relative standard deviation = 4.17%) for whole blood.

### Laboratory methods.

Parasitemia and hematocrit were determined every 6 hours until parasite clearance, and then on days of follow-up. Malaria blood films were stained with Giemsa solution, and parasites counted against 500 white blood cells or against 1,000 red blood cells. Two qualified microscopists read all the slides independently, and parasite densities were calculated by averaging the two counts. A glucose-6-phosphate dehydrogenase (G6PD) deficiency rapid diagnostic test, the CareStart^©^ (Access Bio, Inc. Somerset, NJ) screening test, was also performed on day 0.

### Follow-up.

Patients were admitted to the communal health stations for at least 48 hours or longer until two consecutive blood smears were negative for parasites. A clinical, microscopy and hematological assessment was performed on day 3 (at 72 hours), day 7, and then once a week until day 63 for follow-up of *P. vivax*. Follow-up activities included clinical assessment, measurement of temperature, hematocrit, parasitological assessment, and alternative treatment in case of treatment failure.

### Outcome measures.

The primary endpoint of this study was the proportion of patients classified as having an adequate clinical and parasitological response during a follow-up period of 63 days. In addition, the proportions of early treatment failures, late clinical failures, and late parasitological failures, as well as the time to treatment failure, were also reported as secondary endpoints. Adequate clinical and parasitological response, early treatment failure, late clinical failure, and late parasitological failure were defined using the same criteria as for the assessment of *P. falciparum* malaria.^21^

Other secondary outcomes included 1) parasite clearance half-life *T*_1/2_, that is, the time for parasitemia to fall by half during the log-linear phase of parasite clearance as defined by the Parasite Clearance Estimator developed by the Worldwide Antimalarial Resistance Network^22^ (note that this estimator has been validated in *P. falciparum* only and hence usage for *P. vivax* is exploratory); 2) parasite clearance time, defined as the time in hours from the first treatment dose to the first of two consecutive parasitemia counts below the detection limit of 20 parasites/μL (patients without documented parasitemia clearance were censored at the time of the last measured positive parasite count); 3) proportion of patients with a parasite clearance time > 48 hours after starting treatment; and 4) fever clearance time, defined as the time in hours from the first treatment dose to the start of the first sustained period of 24 hours without fever (i.e., temperature < 37.5°C).

### Sample size.

The trial was powered to demonstrate the non-inferiority of DHA-PPQ as compared with the standard of care of chloroquine treatment with respect to the primary endpoint, the proportion of patients with an adequate clinical and parasitological response on day 63. Assuming an identical adequate clinical and parasitological response probability of 90% on day 63 in both treatment groups, a non-inferiority margin of 10%, a one-sided significance level of 2.5%, and an 80% power, a minimum of 142 patients per study arm were required. To accommodate losses to follow-up and protocol violations, the sample size was increased by 16%, giving a target sample size of 330 patients (in total).

### Statistical analysis.

Following World Health Organization guidelines,^20^ the probability of adequate clinical and parasitological response on day 63 was calculated based on the Kaplan–Meier method including all randomized patients and, additionally, as a proportion for the subset of patients with complete follow-up until day 63. A two-sided Wald-type 95% confidence interval (CI) for the difference in adequate clinical and parasitological response probabilities was created based on the estimates and corresponding standard errors (calculated according to Greenwood's formula) for the Kaplan–Meier analysis and on a normal approximation of the absolute risk difference for the proportion analysis, respectively. In both cases, corresponding *P* values for testing superiority of DHA-PPQ were also reported. The parasite clearance half-life was compared between the two arms using the *t* test. Parasite and fever clearance times were summarized with Kaplan–Meier estimates of median and interquartile range (IQR), and comparisons between groups were based on the score test of a Cox-regression analysis with the treatment arm as the only covariate. All endpoints were reported and compared on the intention-to-treat (ITT) population containing all randomized patients. The primary endpoint was additionally reported for the per-protocol population, which excluded all patients who were withdrawn or lost to follow-up at any time point during the 63-day follow-up. The reported standard estimates, two-sided 95% CIs, and tests do not account for the fact that the trial was stopped early. All analyses were performed with the statistical software R3.2.0 (R Foundation for Statistical Computing, Vienna, Austria).

### Ethical approval.

`The study was approved by the Ethical Committee of the Ho Chi Minh Institute of Malaria-Parasitology-Entomology and the Oxford University Tropical Research Ethics Board. Clinicaltrial.gov registration number: NCT01887821.

## Results

When the study started in early 2013 radical treatment was not routinely provided, as primaquine was not routinely available. Availability improved substantially during the trial and new national guidelines on malaria treatment were introduced. As a result the trial steering committee decided to terminate the trial and provide radical treatment to all patients with *P. vivax* malaria.

**Figure 1.**
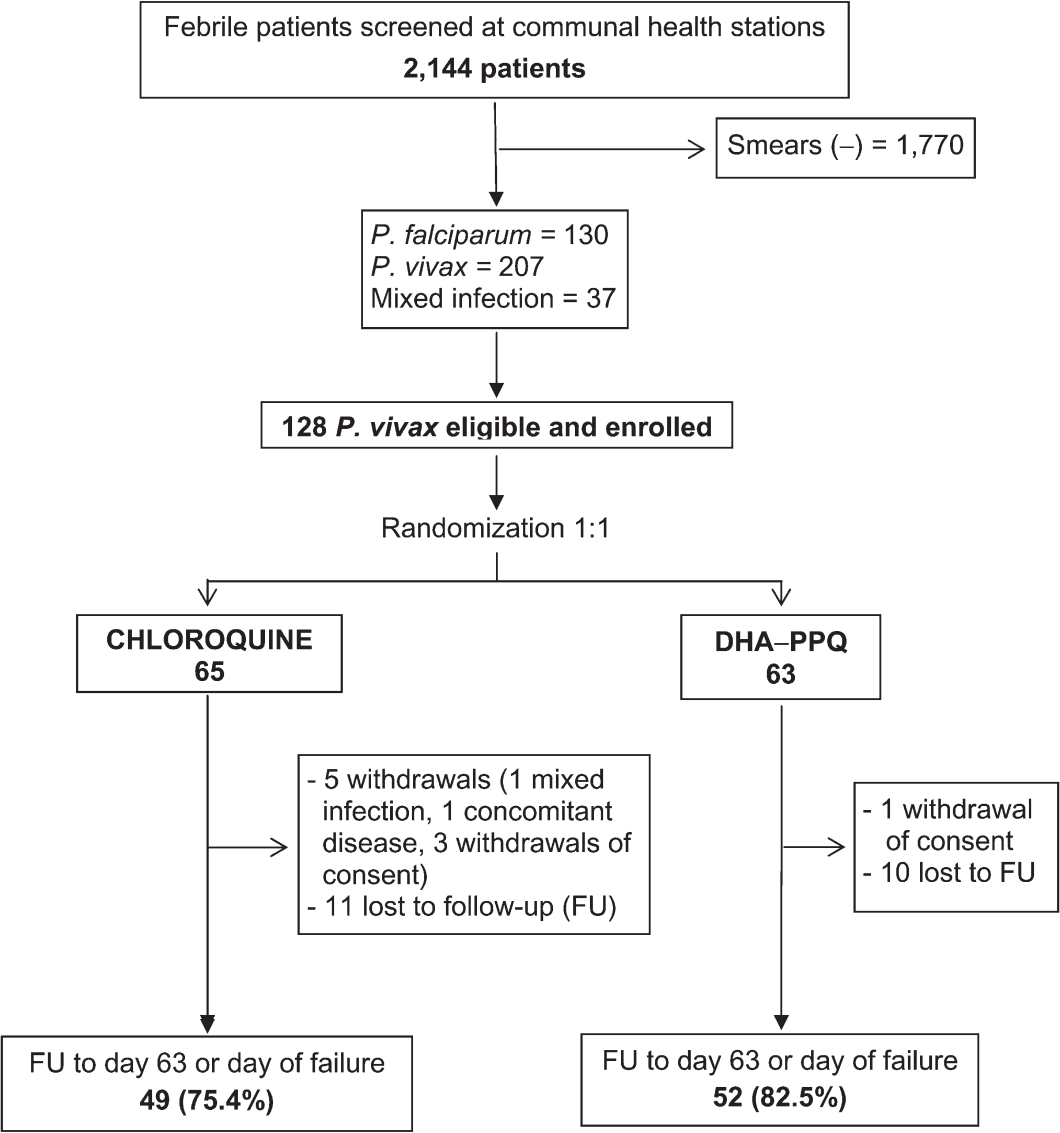
Study flow diagram.

The trial started in February 2013 and stopped in November 2014 after 128 of 330 planned patients had been enrolled. The proportion of patients with positive smears among those presenting to the health stations during the above period was 374/2,144 (17.4%). There were 65 patients enrolled in the chloroquine arm and 63 in the DHA-PPQ arm. Follow-up to day 63 or day of failure (per-protocol analysis) was achieved in 49/65 (75.4%) patients treated with chloroquine and in 52/63 (82.5%) patients given DHA-PPQ (Figure 1

). Most patients recruited into the study were adults (104/128, 81.3%) and males (109/128, 85.2%). The baseline characteristics of the 128 randomized patients are presented in Table 1.

**Figure 2.**
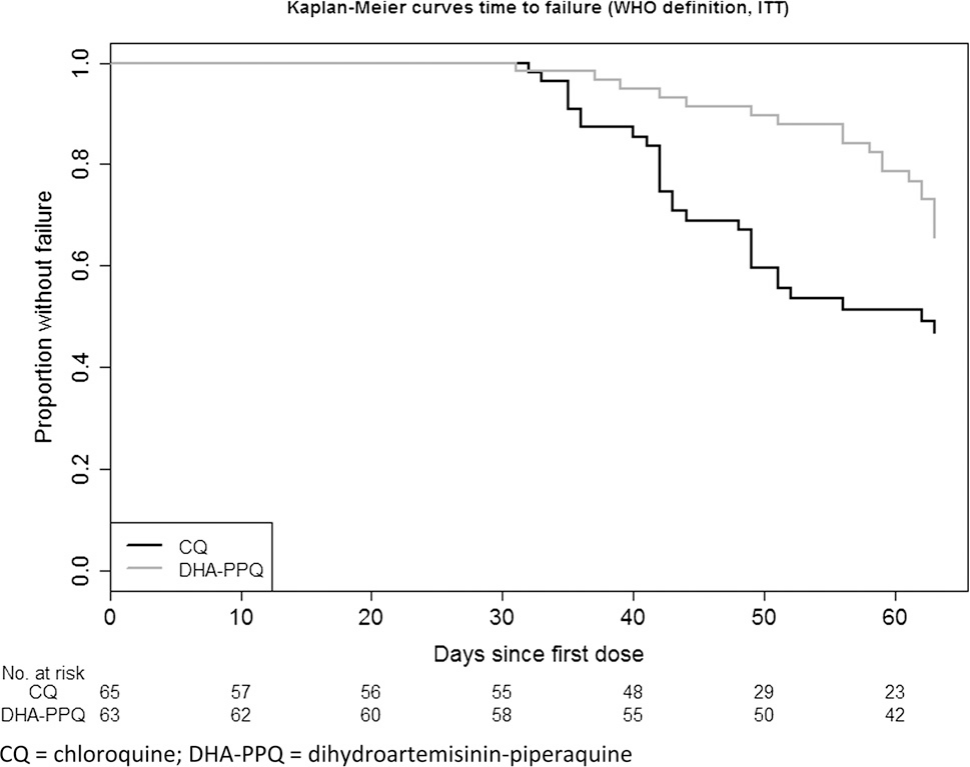
Kaplan–Meier curves of time to failure by treatment groups.

The estimated probabilities of adequate clinical and parasitological response were 47% in the chloroquine arm and 66% in the DHA-PPQ arm in the ITT analysis. In per-protocol analysis, the corresponding proportions were 43% and 63%, respectively (Table 2). The 95% CI of difference in adequate clinical and parasitological response rates in both analyses excluded a difference of 10% or more in favor of the chloroquine arm confirming the non-inferiority of DHA-PPQ compared with chloroquine. Two-sided *P* values indicated borderline superiority of DHA-PPQ (*P* = 0.051 ITT analysis and *P* = 0.034 per-protocol analysis). No early treatment failures were observed in either treatment arm. Late clinical failures and late parasitological failures occurred in 10 and 18 patients in the chloroquine arm compared with five and 14 in the DHA-PPQ arm. The earliest failure events were one late parasitological failure in the DHA-PPQ arm on day 28 and one late clinical failure on day 33 in the chloroquine arm. The rate of recurrent infections was significantly lower in the DHA-PPQ group (hazard ratio = 0.47 [95% CI = 0.26 to 0.83]; *P* = 0.009) (Figure 2

).

**Figure 3.**
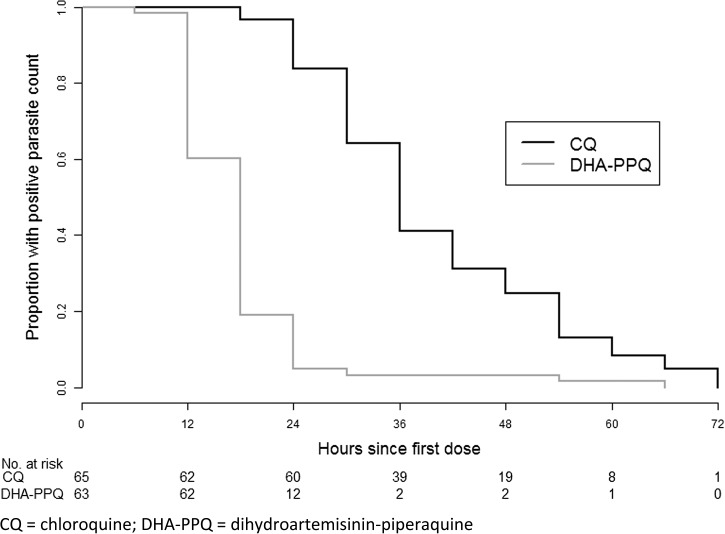
Kaplan–Meier curves of parasite clearance time by treatment group.

The mean parasite clearance half-lives were 3.98 hours in the chloroquine arm and 1.80 hours in the DHA-PPQ arm (*P* < 0.001). The median (IQR) parasite clearance time were 36 (30, 48) hours in the chloroquine arm and 18 (12, 18) hours in the DHA-PPQ arm (Figure 3

). The parasite clearance time was significantly shorter in the DHA-PPQ arm (hazard ratio = 5.55 [95% CI = 3.74 to 8.24]; *P* < 0.001). The proportions of patients with parasite clearance time > 48 hours were 25% in the chloroquine arm and 3% in the DHA-PPQ arm (*P* < 0.001). Median (IQR) fever clearance time were 24 (6, 36) hours in the chloroquine arm and 12 (12, 18) hours in the DHA-PPQ arm. Fever clearance was significantly faster in the DHA-PPQ arm (hazard ratio = 1.53 [95% CI = 1.07 to 2.18]; *P* = 0.02).

Table 2 presents the summary of primary and secondary endpoints by treatment group (ITT population).

### Additional laboratory exploratory analyses.

Among 128 enrolled patients, there was one case of G6PD deficiency in the chloroquine arm, a male of the S'Tiêng ethnic minority.

Among blood samples collected on day 0 at enrollment, 9/60 (15%) patients had detectable chloroquine and MCQ at low concentrations in whole blood (lowest = 5 ng/mL; highest = 34 ng/mL). By day 28, only 4/54 patients had whole blood chloroquine and MCQ concentrations ≥ 100 ng/mL (highest value = 140 ng/mL), the putative minimal effective concentration for chloroquine-sensitive *P. vivax*.^23^ On the day of recurrent parasitemia, none of the patients had a chloroquine and MCQ concentration ≥ 100 ng/mL in whole blood (highest level detected = 52 ng/mL). Table 3 presents measurements of chloroquine and MCQ in whole blood on days 7, 28 and the day of recurrent parasitemia.

The treatments were well tolerated, and no serious adverse events were recorded in either arm.

## Discussion

In Vietnam, chloroquine has been the drug of choice for *P. vivax* malaria for over 50 years. Few studies have been conducted to assess the efficacy of chloroquine against *P. vivax*. Data from 1995 in southern, central Vietnam showed that of the evaluable *P. vivax* patients, 23/23 (100%) had sensitive infections,^10^ but in another 28-day study conducted from 1997 to 2000 in southern Vietnam, recurrent parasitemia was reported in 18/113 (16%) patients, suggesting the emergence of chloroquine resistance.^12^ In a more recent 28-day study of 64 *P. vivax* cases, also conducted in central Vietnam (2009), there were 9.4% recurrences.^13^

Our 63-day study at two sentinel sites in an area of moderately high malaria transmission in southern Vietnam confirmed that both chloroquine and DHA-PPQ are well-tolerated and efficacious treatments for *P. vivax* malaria. DHA-PPQ was definitely not inferior to chloroquine and in some therapeutic aspects was superior. Fever and parasite clearance were more rapid, and overall recurrence rates were lower.^5^,^7^,^16^,^17^

Experience from the use of DHA-PPQ in the treatment of *P. falciparum* in Vietnam over many years showed that this is a safe and well-tolerated regimen. If DHA-PPQ is accepted for the treatment of both *P. falciparum* and *P. vivax*, the procurement and distribution of antimalarials of the national malaria control/elimination program will be simplified.

There were 47 “late treatment failures” in this study which, except for one recurrent parasitemia on day 28 in the DHA-PPQ arm, happened after day 35 when blood concentrations of both drugs had declined. Primaquine was not given in this study, and so it is difficult to determine the nature of these recurrences (i.e., whether they were relapses from liver hypnozoites, recrudescences caused by parasites surviving in the blood as a result of inadequate or ineffective treatment or reinfections). According to Baird, recurrent parasitemia within 35 days of chloroquine therapy supports a provisional diagnosis of resistance.^23^ The earliest case of recurrent parasitemia in the chloroquine arm occurred on day 33 with corresponding chloroquine and MCQ concentrations of 10.7 and 8.6 ng/mL in the patient's whole blood and plasma, respectively. Among recurrent parasitemias in the chloroquine arm, 17/28 (61%) occurred from day 42 to day 49 suggesting these recurrences were probably relapses from latent hypnozoites, as elsewhere in the southeast Asia region.^24^ The lower rate of recurrences in the DHA-PPQ arm may reflect more potent suppression of relapses or reinfections during the follow-up period.

Regarding G6PD status among enrolled patients, only one case of deficiency was recorded in a male of S'Tiêng ethnic minority. Confirmation of G6PD status using the standard enzyme-linked immunosorbent assay method in our study population is in progress and results will be reported later. Previous studies have shown that G6PD deficiency determined by the methylene blue reduction test and by G-6-PDH kit (Sigma diagnostics, Dorset, United Kingdom) was 8.7% (23/266) in Kinh ethnic people and 14% (36/258) in S'Tiêng people, respectively,^25^ although rates in *P. vivax* malaria are often lower as G6PD deficiency provides some protection. All the patients in our study received 14-day primaquine on day 63 or day of recurrent parasitemia (except the one G6PD deficiency case mentioned above), and there was no hemoglobinuria or other adverse effects.

Taken together, these findings suggest that chloroquine is still an effective treatment of *P. vivax* malaria but that DHA-PPQ offers clinical and operational advantages.

In recent years, benefits and disadvantages of using ACTs, especially combinations with partner drugs that have long half-lives (DHA-PPQ, artesunate–mefloquine), have been reviewed.^16^,^17^,^26^ In addition to prompt clinical and parasitological responses with shorter fever clearance times and parasite clearance times, ACTs that provide very rapid reduction in parasite biomass, including gametocytemia, may suppress resistant strains more effectively than chloroquine alone, and consequently, possess a transmission blocking potential.^26^ Operationally, using ACTs in remote areas in malaria-endemic regions or in the private sector where the correct differential parasitological diagnosis is limited may help to avoid wrong drug choices and consequent severe complications. In terms of cost-effectiveness, an additional cost linked with using ACTs for both *P. falciparum* and *P. vivax* malaria may be a concern for policy makers, but in Vietnam the cost difference would not be large because DHA-PPQ is locally produced. In any case, the clinical and operational benefits of a single simple effective treatment would outweigh the disadvantages.

To conclude, chloroquine remains efficacious for the treatment of *P. vivax* malaria in southern Vietnam. DHA-PPQ, not inferior to chloroquine in efficacy, is an alternative treatment with many advantages over chloroquine: shorter parasite clearance half-life, parasite clearance time, and fever clearance time and lower proportion of parasite clearance time > 48 hours.

## Figures and Tables

**Table 1:** Baseline characteristics by treatment group (ITT population)

	Chloroquine (*N* = 65)	DHA-PPQ (*N* = 63)
Age	21 (15, 32)	23 (18, 32)
Sex		
Female	11 (17%)	8 (13%)
Male	54 (83%)	55 (87%)
Ethnicity		
Kinh	24 (37%)	26 (41%)
S'Tiêng	13 (20%)	17 (27%)
Other ethnicities	28 (43%)	20 (32%)
Previous malaria episodes	34 (52%)	27 (43%)
Febrile on admission	56 (86%)	55 (87%)
Temperature (°C)	38.6 (37.7, 40.0)	39 (37.9, 39.7)
Height (cm)	160 (153, 167)	162 (160, 169)
Weight (kg)	51 (45, 58)	52 (47, 58)
Parasitemia at enrollment (parasite/μL)	15,206 (4,241.5, 22,534)	10,362 (3,490, 20,101)
Hematocrit (%)	40 (39, 44)	42 (39, 45)

DHA-PPQ = dihydroartemisinin–piperaquine; ITT = intention-to-treat. Summary statistic is absolute count (%) for categorical variables and median (interquartile range) for continuous data. Parasitemia at enrollment was missing for three chloroquine patients. Hematocrit was missing for two chloroquine and one DHA-PPQ patients. There were no other missing data for any of the reported characteristics.

**Table 2:** Summary of primary and secondary endpoints by treatment group (ITT population)

Characteristics	Chloroquine (*N* = 65)	DHA-PPQ (*N* = 63)	Overall comparisons: estimate (95% CI); *P* value
Treatment outcomes until day 63 (WHO definition)			
ACPR*	21 (32%)	33 (52%)	
Early treatment failure	0 (0%)	0 (0%)	
Late clinical failure	10 (15%)	5 (8%)	
Late parasitological failure	18 (28%)	14 (22%)	
Withdrawn or lost from follow-up	16 (25%)	11 (17%)	
Probability of ACPR			
Kaplan–Meier analysis (ITT)†	47% (33%, 61%)	66% (53%, 79%)	Difference in probabilities: 19% (0–37%); *P* = 0.051‡
Proportion (“per protocol”)	21/49 (43%)	33/52 (63%)	Difference in probabilities: 21% (2–40%); *P* = 0.034‡
Clearance half-life *T*_1/2_ (hours)§			
Mean; median (IQR)	3.98; 3.68 (2.76, 4.7)	1.80; 1.65 (1.3, 1.94)	Difference in mean half-life: −2.18 (−2.66 to −1.69); *P* < 0.001∥
Parasite clearance time (PCT100)¶			
Median (IQR) (hours)†	36 (30, 48)	18 (12, 18)	Hazard ratio of time to clearance: 5.55 (3.74–8.240); *P* < 0.001
Probability of PCT100 > 48 hours†	25% (14%, 36%)	3% (0%, 8%)	Difference in probabilities: −22% (−33% to −10%); *P* < 0.001
Fever clearance time			
Median (IQR) (hours)†	24 (6, 36)	12 (12, 18)	Hazard ratio of time to clearance: 1.53 (1.07 to 2.180; *P* = 0.02

ACPR = adequate clinical and parasitological response; CI = confidence interval; DHA-PPQ = dihydroartemisinin–piperaquine; ITT = intention-to-treat; IQR = interquartile range.

* Four patients in the DHA-PPQ were recorded on the database as having a late parasitological failure that occurred after day 63, and they were treated as having an ACPR as per the predefined day 63 cutoff. If they would be treated as failures instead, the proportion of ACPR in the DHA-PPQ group would decline to 29/52 (56%) and the difference in proportions (“per protocol”) would be 13% (−6% to 32%); *P* = 0.19.

† Kaplan–Meier estimates of median (IQR) or probability (95% CI).

‡ Two-sided *P* values correspond to tests for a difference not for non-inferiority of DHA-PPQ.

§ Half-lives could not be estimated for four patients on chloroquine and three patients on DHA-PPQ.

∥ CI and *P* value based on *t* test.

¶ Three patients on chloroquine did not have any parasite counts recorded and were considered censored at time 0.

**Table 3:** Chloroquine-MCQ concentrations in whole blood (ng/mL)

Day of measurement	Chloroquine-MCQ concentrations in whole blood (ng/mL)				
	*n*	Mean (SD)	Median	Lowest value	Highest value
Day 7	58	537.32 (202.35)	520.55	246.63	1,280.41
Day 28	51	55.49 (26.19)	46.14	23.06	139.67
Day of recurrence of parasitemia	28	21.13 (12.69)	20.45	0.00	51.96

MCQ = monodesethylchloroquine; SD = standard deviation.
